# Association of Genomic Instability with HbA1c levels and Medication in Diabetic Patients

**DOI:** 10.1038/srep41985

**Published:** 2017-02-02

**Authors:** Annemarie Grindel, Helmut Brath, Armen Nersesyan, Siegfried Knasmueller, Karl-Heinz Wagner

**Affiliations:** 1 Department of Nutritional Sciences, Emerging Field Oxidative Stress and DNA Stability, University of Vienna, Vienna, Austria; 2 Research Platform Active Ageing, University of Vienna, Vienna, Austria; 3 Diabetes Outpatient Clinic, Health Centre South, Vienna, Austria; 4 Institute for Cancer Research, Medical University Vienna, Vienna, Austria

## Abstract

Diabetes Mellitus type 2 (DM2) is associated with increased cancer risk. Instability of the genetic material plays a key role in the aetiology of human cancer. This study aimed to analyse genomic instability with the micronucleus cytome assay in exfoliated buccal cells depending on glycated haemoglobin (HbA1c) levels and medication in 146 female DM2 patients. The occurrence of micronuclei was significantly increased in DM2 patients compared to healthy controls. Furthermore, it was doubled in DM2 patients with HbA1c > 7.5% compared to subjects with HbA1c ≤ 7.5%. Positive correlations were found between micronuclei frequencies and HbA1c as well as fasting plasma glucose. Patients under insulin treatment showed a two-fold increase in micronuclei frequencies compared to subjects under first-line medication (no drugs or monotherapy with non-insulin medication). However, after separation of HbA1c (cut-off 7.5%) only patients with severe DM2 characterised by high HbA1c and insulin treatment showed higher micronuclei frequencies but not patients with insulin treatment and low HbA1c. We demonstrated that the severity of DM2 accompanied by elevated micronuclei frequencies predict a possible enhanced cancer risk among female DM2 patients. Therapy, therefore, should focus on a strict HbA1c control and personalised medical treatments.

Depending on the duration and the severity of DM2, lifestyle changes are initially advised and followed by first-line therapeutics such as metformin, combined with a second or third non-insulin medication and completed with insulin treatment^1^,^2^,^3^. In spite of all efforts, many DM2 patients fail to achieve a general HbA1c goal of <7.0%^4^. Glycaemic control is absolutely indispensable in order to prevent macro- and microvascular complications and cardiovascular events in DM2^5^. High blood glucose not only affects the vascular system but recent evidence suggests that HbA1c levels are associated with cancer risk^6^,^7^. Keen interest persists on medical treatments influencing cancer risk in DM2, however, results are still controversial^8^.

Tumorigenesis and cancer initiation are promoted by genomic instability which occurs through alterations in the genome during cell division^9^,^10^. A suitable approach to assess genomic instability is the micronucleus cytome assay in exfoliated buccal cells - a non-invasive and effective method to appraise several biomarkers regarding nuclear anomalies^11^. Thereby, the most important biomarker is the number of micronuclei (MN) which are formed as a consequence of structural and numerical chromosomal aberrations. Other parameters are nuclear buds (NB) reflecting gene amplification events, binucleated cells (BNC) indicating disturbed mitosis and basal cells reflecting the proliferation of epithelia. Cytotoxic effects are represented by karyorrhexis (KR), karyolysis (KL), condensed chromatin (CC) and pyknosis (P)^11^,^12^.

The aim of this study was to analyse genomic instability in DM2 patients depending on HbA1c-levels and medical treatment in order to predict a possible cancer risk.

## Results

### Characteristics of the study population separated by HbA1c groups (HbA1c ≤ 7.5%|HbA1c > 7.5%)

While there was no difference in age, patients with HbA1c > 7.5% showed significantly higher BMI (33.7|36.4 kg/m^2^), waist circumference (102.8|107.7 cm), systolic blood pressure (BP) (137.7|145.6 mmHg) and longer diabetes duration (13.6|15.2 y). HbA1c (6.9|8.7%) and fasting plasma glucose (FPG) (7.9|10.1 mmol/L) were significantly increased in the high HbA1c group while fasting insulin, C-peptide and homeostatic model assessment-insulin resistence (HOMA-IR) did not differ (Table 1).

### Genomic damage in DM2 patients depending on HbA1c

While proliferation of epithelia measured by basal cell frequency was not different between the HbA1c groups, genomic damage parameters were significantly higher in the HbA1c >7.5% group. The number of MN cells was increased in this group by +50.8% (p = 0.002), total number of MN by +119.3% (p = 0.000), and KL by +41.5% (p = 0.000) compared to HbA1c ≤ 7.5% group (Table 2). A trend in higher NB frequencies was shown for the high HbA1c group. However, when broken eggs (BE) as part of NB were considered separately, frequencies were significantly increased in the HbA1c > 7.5% group (HbA1c ≤ 7.5%: 1.04 ± 0.7‰/HbA1c > 7.5%: 1.44 ± 0.8‰; p = 0.002).

**Figure 1:**
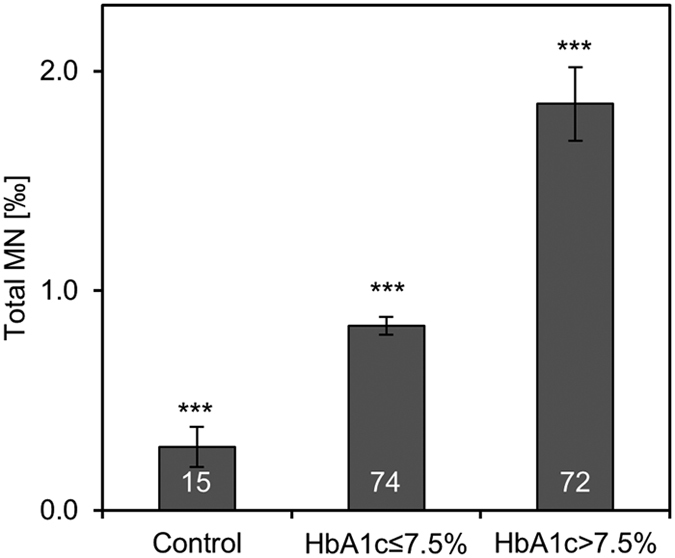
Differences of MN between HbA1c ≤ 7.5%, HbA1c > 7.5% and healthy controls. Control group comprised 15 female healthy controls which were described previously^13^. General characteristics of controls can be found in Supplementary Table 1. Bars show means and standard errors. White numbers in bars indicate the number of patients. Differences between the groups were analysed with Kruskal-Wallis test with pairwise comparisons. All groups were significantly different to each other with p < 0.001, indicated with ***.

The inclusion of a healthy female control group (n = 15) with MN frequency of 0.29 ± 0.4‰ showed that DM2 patients had significantly higher amounts MN occurrence (Fig. 1). The control group was part of a recently performed study in our lab and general characteristics can be found in Supplementary Table 1^ ^13.

**Figure 2:**
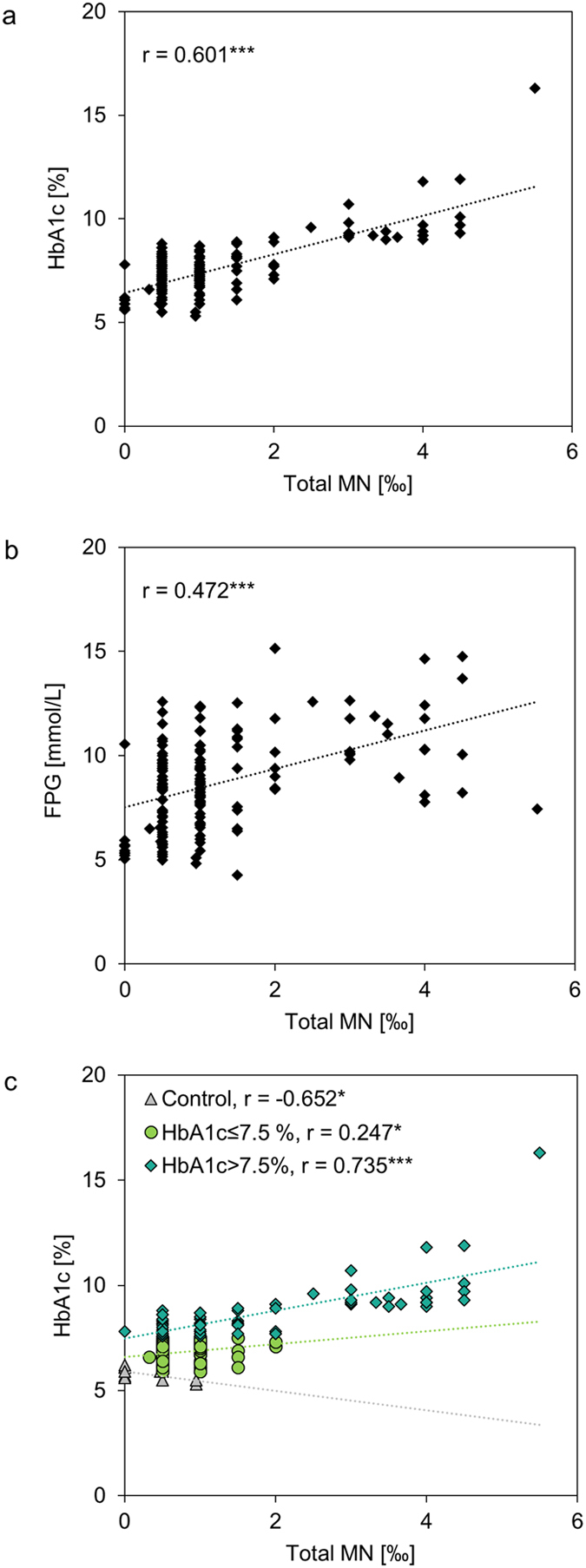
Spearman correlation analyses were performed for total MN and HbA1c (**a**) and for total MN and FPG (**b**) for all DM2 subjects plus healthy controls (n = 161). Correlation analyses for controls (n = 15) and patients with either HbA1c ≤ 7.5% (n = 74) or HbA1c > 7.5% (n = 72) are presented for HbA1c with total MN (**c**). Control group comprised 15 female healthy controls which were described previously^13^. r, spearman correlation coefficient; * indicates significance with p < 0.05 and *** for p < 0.001.

Linear correlations occurred between total MN and HbA1c with r = 0.601 (p = 0.000) and between total MN and FPG with r = 0.472 (p = 0.000). Interestingly, patients with HbA1c > 7.5% showed stronger correlations between HbA1c and total MN (r = 0.735; p = 0.000) compared to subjects with HbA1c ≤ 7.5% (r = 0.247; p = 0.034) (Fig. 2).

### Medical Treatment and creation of Med groups

All patients were under medical observation with diabetes treatment including lifestyle advices alone or in combination with diabetes drugs. In total, 33 different diabetes drugs were taken by the patients in monotherapy or different combinations. For a detailed summary see Supplementary Figure 1. Ongoing, three medication groups were created for further statistical analyses: Med A included patients under non-insulin monotherapy or lifestyle advices alone (n = 18); Med B included patients under non-insulin combination therapy (n = 72); Med C included patients under insulin treatment (with or without other non-insulin medication) (n = 56).

### MN frequencies depending on Med groups

**Figure 3:**
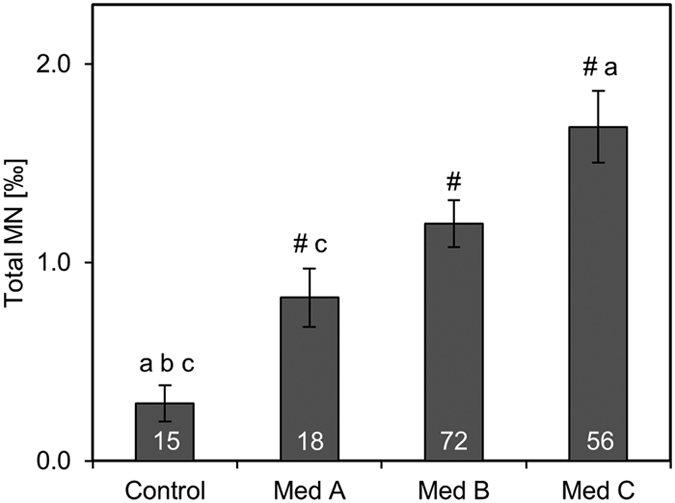
MN frequencies depending on Med groups. Total MN frequencies were assessed in controls (n = 15) and patients of three medication groups: Med A: no medication or non-insulin monotherapy; Med B: non-insulin combination therapy; Med C: insulin medication (with or without other non-insulin medication).Control group comprised 15 female healthy controls which were described previously^13^. Bars show means and standard errors. White numbers in bars indicate the number of patients. Differences between the groups were analysed with Kruskal-Wallis test with pairwise comparisons. Significance was assumed with p < 0.05 and is indicated with # (difference to control), a (difference to Med A), b (difference to Med B), c (difference to Med C).

**Figure 4:**
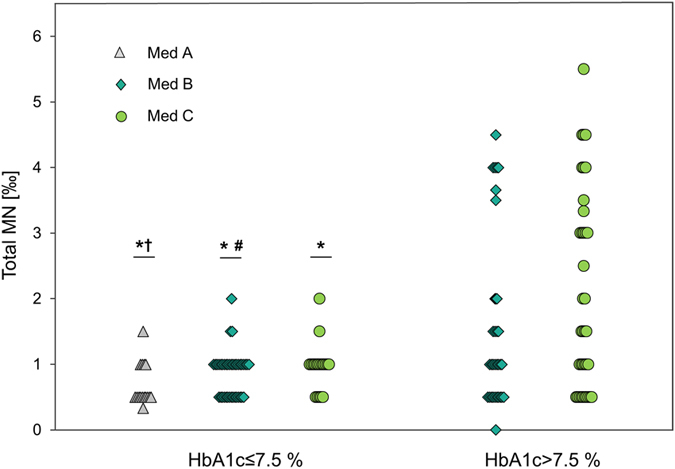
Distribution of MN frequencies depending on Med groups and HbA1c (cut-off 7.5%). Med A: no medication or non-insulin monotherapy; Med B: non-insulin combination therapy; Med C: insulin medication (with or without other non-insulin medication). Each dot represents MN frequency of one patient. Med A within HbA1c > 7.5% was excluded from statistics due to only two cases. Differences between the groups were analysed with ANCOVA, diabetes duration and BMI as covariates. *represents significant difference to Med C of HbA1c > 7.5% with p < 0.01. ^#^represents significant difference to Med B of HbA1c > 7.5% with p < 0.05. ^†^indicates trend of difference to Med B of HbA1c > 7.5% with p < 0.1.

Patients of Med C had 104% more MN compared to subjects of Med A. Additionally, all Med groups showed higher MN frequencies compared to healthy controls (p < 0.05; Fig. 3). In dependency of HbA1c, patients with HbA1c > 7.5% and Med C had significantly more MN compared to all Med groups with low HbA1c (Med A: p = 0.001; Med B: 0.000; Med C: p = 0.003). Additionally, patients in Med B and HbA1 > 7.5% showed increased MN frequencies compared to Med B (p = 0.033) and a trend to Med A (p = 0.058) of HbA1c ≤ 7.5% group. Notably, there was also a broader distribution of MN in the high HbA1c group than in the low HbA1c group (Fig. 4).

### PCA and bi-clustering

**Figure 5:**
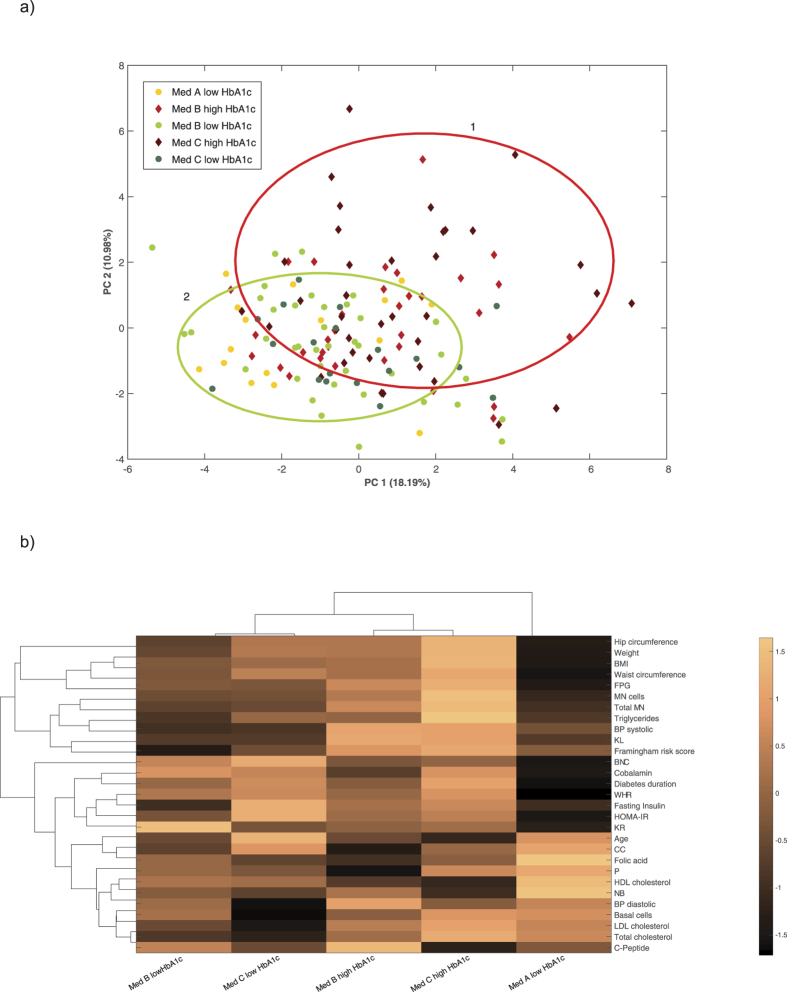
COVAIN results of PCA and bi-clustering of the 5 groups, divided by HbA1c and medication (Med A, B, C for HbA1c ≤ 7.5% and Med B, C for HbA1c > 7.5%). Med A: no medication or non-insulin monotherapy; Med B: non-insulin combination therapy; Med C: insulin medication (with or without other non-insulin medication). Med A for HbA1c > 7.5% was excluded from statistics because of only 2 cases. (**a**) PCA plot shows two overlapping clusters representing the high HbA1c Med groups (red circle 1) and the low HbA1c Med groups (green circle 2). (**b**) Heat map shows bi-clustering of the 5 groups with contributing variables. A distinct grouping of Med B&C for either HbA1c ≤ 7.5% or HbA1c > 7.5% and a separate standing of Med A resulted. For detailed information about the differences between the 5 groups see Supplementary Table 2.

To elucidate how the 5 different groups, divided by HbA1c and medication (Med A, B, C for HbA1c ≤ 7.5% and Med B, C for HbA1c > 7.5%) are linked based on anthropometrics, lipid metabolites, vitamins and genomic instability, a more in-depth principle component analysis (PCA) and bi-clustering using COVAIN was performed^14^. The main separation appeared to be between the HbA1c groups. The PCA plot showed an expanded distribution of patients with high HbA1c levels while the low HbA1c Med groups are clustered closer together (Fig. 5a). Bi-clustering resulted in the grouping of Med B&C with either HbA1c ≤ 7.5% or HbA1c > 7.5% and a separate standing of Med A with HbA1c ≤ 7.5% (Fig. 5b). For detailed information about the differences between the groups see Supplementary Table 2.

## Discussion

This explorative study aimed to determine a possible association between genomic instability as a precursor in cancer initiation with HbA1c and medication in female DM2 patients. Considering HbA1c, the findings clearly show the strong association of genomic damage with blood glucose control in DM2 patients. Subjects with HbA1c > 7.5% showed 2-fold higher MN frequencies compared to the ones with lower HbA1c values. Additionally, a linear correlation was detected between HbA1c and scored MN, which was particularly pronounced in patients with HbA1c > 7.5%. These results are supported by two recent publications which reported higher MN frequencies compared to healthy controls and an HbA1c-dependent increase of genomic instabilities^13^,^15^. In addition, several studies showed an increased cancer risk in diabetes patients compared to respective controls^6^,^16^,^17^,^18^,^19^,^20^,^21^,^22^,^23^. Moreover, two recent studies were able to show the association between the overall cancer risk (excluding liver and prostate cancer) and HbA1c levels within the diabetic but also the non-diabetic HbA1c range^6^,^7^.

HbA1c levels reflect the average plasma glucose within the last eight to twelve weeks^24^ and appear to be correlated to genomic damage as well as to cancer risk. Blood glucose is dependant of the severity and duration of DM2 as well as successful diabetes treatment. We therefore tested our second aim in a more in-depth statistical analysis regarding genomic instabilities and medication. As shown in Supplementary Figure 1, the study population was quiet heterogeneous in their medical treatments, especially due to different combinations. Therefore, the comparison of single drug classes was not possible with our study size. We created three Med groups according to the guidelines of the American Diabetes Association and the European Association for the Study of Diabetes which state that therapeutic treatment start with diet and exercise advice followed by first-line drugs (usually metformin), combined with second or third non-insulin medication and completed with insulin treatment^1^,^2^,^3^. In this study patients under insulin treatment had 2-fold higher MN frequencies compared to patients under first-line medication.

To our knowledge no other study has ever analysed the link between diabetes treatment and genomic instability measured with the MN cytome assay. However, many studies dealt with the topic of cancer risk and medication of DM2 in the last decade predicting a general assumption which reflects our MN results: insulin sensitizer, such as metformin, lower cancer risk while drugs which increase insulin (endogenously or exogenously) increase cancer risk^8^,^25^. Especially exogenous insulin therapy was associated with increased all-cause mortality and cancer^26^ which might be explained by its possibility to bind and activate the structurally similar insulin-like growth factor-1 leading to cell growth and differentiation^27^,^28^. However, it should be taken into account that insulin in DM2 is only prescribed when other medications fail to achieve treatment goals^3^. Hence, a time-related bias occurs which often is not statistically considered. Indeed, a retrospective database analysis which compensated for this bias did not find any effect of different diabetes treatments on cancer incidences^29^.

It remains unclear if the treatments themselves or the severity of hyperglycaemia, longer diabetes duration and secondary metabolic issues influences cancer incidence. We demonstrated that only the patients with severe DM2, characterized by HbA1c > 7.5% and insulin treatment had significantly higher MN frequencies compared to subjects with HbA1c ≤ 7.5% off all Med groups. This indicates that insulin per se does not lead to increased genomic instability but that patients, who cannot control their HbA1c properly, even with insulin injections, are in a weak genomic state. Supporting this, PCA and bi-clustering resulted in grouping according to HbA1c values rather than Med groups, suggesting that HbA1c-control is more important to stay a healthier DM2 course independently of the medication.

It should be considered, that the occurrence of MN in exfoliated buccal cells is not yet a validated cancer risk marker. However, the MN cytome assay in exfoliated buccal cells is used extensively for measuring genotoxicity. It is well documented that MN occurrence in exfoliated buccal cells is elevated in individuals who already suffer from cancer or who have increased cancer risks due to occupational and environmental exposures or due to cancer associated diseases (for review see refs 30,31). Thereby a recent meta-analysis found higher MN frequencies not only in patients with oral and neck cancer (meta MR 2.4) and leukoplakia (meta MR 1.9) but also in patients with other tumors (meta MR 2.0)^30^. This could indicate that the MN frequency reflect also genomic instabilities of other organs. Unfortunately, there is not yet data from long lasting studies addressing the direct link of elevated MN occurrence in exfoliated buccal cells and a later cancer disease. However, it was shown in comprehensive statistical analyses that MN in lymphocytes are a predictive biomarker for cancer risks in humans^32^, and that the systematic comparison of MN frequencies between buccal cells and lymphocytes resulted in a clear linear correlation^33^. Additionally, more than 90% of all human cancers are of epithelial origin^34^ which supports the assumption that epithelial cells may be suitable indicators for the detection of elevated cancer risks^31^. Taken all the evidence into account, there seem to be a connection between elevated MN of exfoliated buccal cells and an enhanced cancer risk.

This explorative study in female DM2 patients found a clear association of HbA1c with genomic instabilities; however, the influence of medication stays questionable. The rather small med groups with only female DM2 patients should be considered critically and we therefore suggest more studies with larger number of patients and further grouping regarding single drug classes and their combinations to assess the influence of DM2 treatment on genomic instabilities and cancer risk. Overall, our results indicate the need for a strict glucose control in DM2 to maintain genome stability and thereby reducing cancer risk. Therefore, regular health checks, early diagnoses, personalized drug prescriptions and increased life-style changes should gain more importance to achieve HbA1c goals.

## Materials and Methods

### Cross-sectional human study

The cross-sectional human study was performed as a cooperation between the large Diabetes Outpatient Clinic in Vienna and the Department of Nutritional Sciences of the University of Vienna in 2014^35^. In total, 154 female DM2 patients were recruited during their biannual health checks by their attending physician six month prior study start. The study included females with an age of 40–90 years, oral anti-diabetes drugs and/or injectables as well as lifestyle approaches as diabetes medication, constant medication in regard to metabolic parameters within the last 4 weeks, HIV negative, non-smoking for at least 1 year and no history of alcoholism within the last 2 years. Further, patients were excluded if pregnant or lactating; reported changes in nutrition, physical activity or weight within the last 4 weeks; had cardiovascular damage with NYHA > III, chronic kidney disease with serum creatinine >2 mg/dl, liver disease with three-fold increase of transaminase values; were on dialyses; or had a history of cancer, stroke or transplantations. Eight patients failed these criteria and were excluded from the study population. The remaining 146 patients were allocated to two groups in respect of their glycaemic control: HbA1c ≤ 7.5% group, n = 74 and HbA1c > 7.5% group, n = 72. Thereby, HbA1c is expressed as % of glycated haemoglobin per total haemoglobin levels. The study was performed in accordance with the Declaration of Helsinki and written consent was received from all participants. The study was approved by the local Ethics Committee of the Medical University of Vienna (EK Nr: 1987/2013) and registered at ClinicalTrials.org (NCT02231736).

In addition to the main study, data from healthy females (n = 15) were taken to serve as control for genomic instabilities. This control group was part of a previously performed study between the Diabetes Outpatient Clinic and the Department of Nutritional Sciences and is explained in detail by Müllner *et al*.^13^.

### Measurements

On the study day, fasting blood samples were taken by venipuncture (Vacuette, K2EDTA, Greiner Bio-one GmbH) and buccal cells were collected by scraping the inside of the cheek with a commercial soft toothbrush after rinsing the mouth with tap water^11^,^13^. Anthropometric measurements were performed lightly dressed without shoes after an overnight fasting period. Weight (scale: selecta 791, Seca), height (stadiometer: model 214, Seca), waist and hip circumference were assessed. BMI was calculated and expressed as m^2^/kg. For blood pressure assessment (Boso medicus control, Bosch + Sohn GmbH), three independent measurements with five minutes break in between were performed and means were calculated. Furthermore, questionnaires regarding the medical history, socio-economic status, nutritional behavior, physical activity, and life quality were completed by the participants.

Diabetes related parameters, including FPG, fasting insulin, HbA1c and C-peptide, as well as lipid parameters (total cholesterol, HDL cholesterol, LDL cholesterol, triglycerides) and vitamins (folic acid and cobalamin) were measured by the laboratory of the Diabetes Outpatient Clinic immediately after blood collection as described before^13^,^36^,^37^. HOMA-IR was calculated with HOMA2 calculator version 2.2.3 (Diabetes Trials Unit, University of Oxford); Framingham risk score was calculated according to D’Agostino *et al*.^38^.

### Micronucleus Cytome Assay

After cell sampling, the head of the toothbrush was placed in a 50 ml flask containing buccal cell buffer prepared according to Thomas *et al*.^11^. The head of the brush was rotated such that the cells got dislodged and released into the suspension. Then the cells were processed as described previously^11^. Briefly, cells were washed three times with buccal cell buffer and centrifuged. Then 5 ml of fresh buffer was added to the cell pellet and the cells were passed into a tube through a 100 μm nylon filter held in a Swinex holder. To prepare the slides, the cell suspension was diluted to 80,000 cells/ml from which 120 μl were added to the respective well of a Shandon Centrifuge. The slides were air dried for 10 min and fixed with chilled (−20 °C) 80% methanol. Ten minutes later, they were placed in glass beakers with 5.0 M HCl at room temperature for 30 min, rinsed with distilled water for 3 min and subsequently stained with Schiff’s reagent (Sigma–Aldrich, Steinheim, Germany) for 90 min, washed 5 min with running tap water and then counterstained with 0.2% (w/v) Light Green (Sigma–Aldrich) for 20 s. From each subject, 2,000 buccal cells were evaluated under a microscope with oil immersion and 1000-fold magnification (Nikon Photophot-FXA, Tokyo, Japan). In the first 1,000 scored cells, basal cells, MN, NB, BNC, CC, KR, P and KL were scored. Following, the frequency of DNA damage biomarkers (MN and NB) was scored in a minimum of 2,000 differentiated cells. All results are presented in ‰. As for NB, a special form exist which was previously described as BE^39^. BE have a similar appearance as NB with a larger bud body (sometimes almost up to the size of the main nucleus). BE and NB were classified together into a single NB category.

### Statistical analyses

Statistical analyses were performed with SPSS (Version 22, IBM). The Kolmogorov-Smirnov test was used for assessing normal distribution. If normal distributed, t-test for independent variables (for 2 groups) or one-way analysis of variance (Anova) with Bonferroni adjustment (more than 2 groups) was performed. For nonparametric data, Mann-Whitney-U test or Kruskal-Wallis test (with pairwise comparisons) were used instead. Analysis of Covariates (ANCOVA) with Bonferroni adjustment for multiple comparisons was applied where necessary. Pearson’s correlation coefficient or Spearman correlation for nonparametric variables was used for correlation analyses. Missing data was excluded from statistics. Principle component analysis (PCA) and bi-clustering analysis were performed with COVAIN toolbox for matlab^14^. To perform bi-clustering the average linkage of Euclidean distance between groups as the metric was used. Therefore, 29 different parameters regarding anthropometrics, lipid metabolism, vitamins and genomic instability were used.

## Additional Information

**How to cite this article**: Grindel, A. *et al*. Association of Genomic Instability with HbA1c levels and Medication in Diabetic Patients. *Sci. Rep.* **7**, 41985; doi: 10.1038/srep41985 (2017).

**Publisher's note:** Springer Nature remains neutral with regard to jurisdictional claims in published maps and institutional affiliations.

## Supplementary Material

Supplementary Information

## Figures and Tables

**Table 1:** Characteristics of study population.

	HbA1c ≤ 7.5% n = 74	HbA1c > 7.5% n = 72	*p-value*
	mean ± SD	mean ± SD	
Age [years]	68.7 ± 9.8	66.2 ± 10	*0.159*
BMI [kg/m^2^]	33.7 ± 7.5	36.4 ± 7.6	***0.021***
Waist circumference [cm]	103 ± 14	108 ± 14	***0.042***
WHR	0.88 ± 0.1	0.89 ± 0.1	*0.403*
BP systolic [mmHg]	138 ± 20	146 ± 19	***0.014***
BP diastolic [mmHg]	81.7 ± 11	82.7 ± 10	*0.578*
FPG [mmol/L]	7.93 ± 1.7	10.1 ± 2.0	***0.000***
HbA1c [%]	6.86 ± 0.5	8.69 ± 1.3	***0.000***
Insulin [pmol/L]	114 ± 94	141 ± 155	*0.321*
C-Peptide [nmol/L]	1.03 ± 0.5	1.02 ± 0.7	*0.405*
HOMA-IR	2.47 ± 2.5	2.70 ± 1.8	*0.179*
Diabetes duration [years]	13.6 ± 8.8	15.2 ± 7.1	***0.044***
Cobalamin [pmol/L]	310 ± 185	299 ± 141	*0.506*
Folic acid [nmol/L]	21.4 ± 13	18.3 ± 10	*0.173*
Ex-smokers [%]	35.1	38.9	*0.640*
Passive smokers [%]	23.0	30.2	*0.302*

Differences between groups were analysed with t-test for independent variables or with Mann-Whitney-U test for nonparametric variables. Significance was assumed with p < 0.05. HbA1c, glycated haemoglobin; SD, standard deviation; BMI, body mass index; WHR, waist-to-hip ration; BP, blood pressure; FPG, fasting plasma glucose; HOMA-IR, homeostatic model assessment-insulin resistance.

**Table 2:** Genomic damage parameters in DM2 patients.

	HbA1c ≤ 7.5% n = 74	HbA1c > 7.5% n = 72	*p-value*
	mean ± SD	mean ± SD	
Basal cells [‰]	8.28 ± 2.9	8.69 ± 3.5	*0.533*
MN cells [‰]	0.80 ± 0.3	1.20 ± 0.8	***0.002***
Total MN [‰]	0.84 ± 0.4	1.85 ± 1.4	***0.000***
BNC [‰]	25.0 ± 10	24.4 ± 9.0	*0.883*
NB [‰]	2.72 ± 1.0	3.08 ± 1.2	*0.055*
KR [‰]	23.8 ± 8.6	23.1 ± 6.7	*0.561*
CC [‰]	25.4 ± 11	23.8 ± 11	*0.278*
KL [‰]	33.1 ± 12	46.9 ± 21	***0.000***
P [‰]	1.28 ± 1.1	1.19 ± 0.8	*0.997*

^1^Differences between groups were analyzed with t-test for independent variables or with Mann-Whitney-U test for nonparametric variables. Significance was assumed with p < 0.05. HbA1c, glycated haemoglobin; SD, standard deviation; MN, micronuclei; BNC, binucleated cells; NB, nuclear buds; KR, karyorrhexis; CC, condensed chromatin; KL, karyolysis; P, pyknosis.
