# Supplementary material for: Association of Genomic Instability with HbA1c levels and Medication in Diabetic Patients
Source: Sci Rep. 2017 Feb 2;7:41985. doi: 10.1038/srep41985 (PMC5288806; doi:10.1038/srep41985)
Supplement: Supplementary Information [file srep41985-s1.pdf]

Supplement

Association of Genomic Instability with HbA1c levels and Medication in Diabetic Patients

Annemarie Grindel, Helmut Brath, Armen Nersesyan, Siegfried Knasmueller, Karl-Heinz Wagner

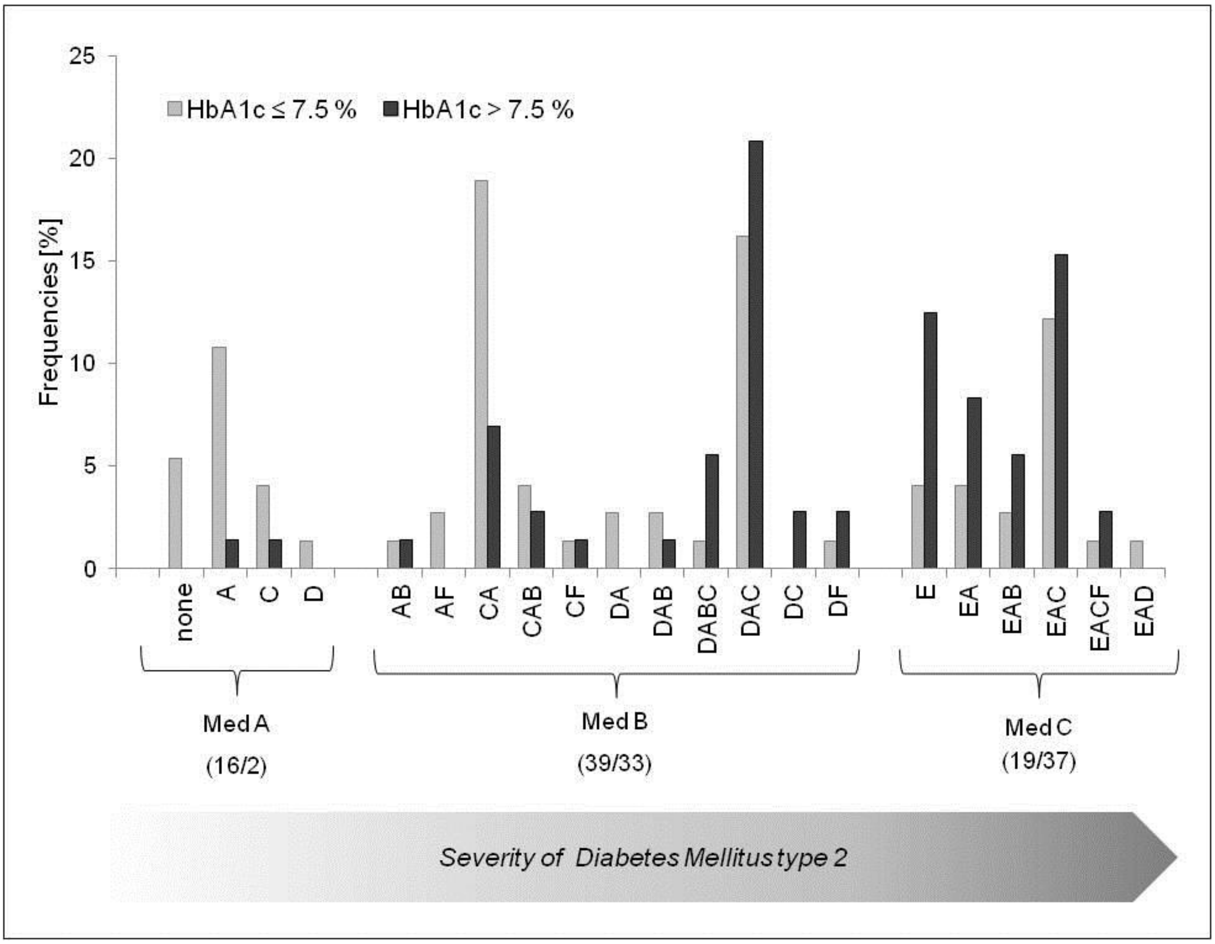

Figure S1 – Frequencies of medical treatment composition

Prescribed medication of all patients was attributed to six major medical drug classes indicated by different letters A-F. A – Metformin; B – SGLT2 inhibitors; C – DPP-4 inhibitors and GLP-1 receptor agonists; D – sulfonylurea and glinides; E – insulin; F – others (including alpha-glucosidase inhibitors, thiazolidinediones). Single letters indicate monotherapy. Several letters indicate the combination of these medical drug classes. Three medication groups were created for ongoing statistical analyses. Med A included patients under no medical treatment and non-insulin monotherapy; Med B included patients under non-insulin combination therapy; Med C included patients under insulin treatment (with or without other non-insulin medication).

Bars show frequencies in percentage. Numbers in brackets indicate total amount of patients in each medication group, while first number indicates patients with HbA1c≤7.5 % and second number indicates patients with HbA1c>7.5 %.

Table S1 – Characteristics of healthy, female control group

	Controls n=15		
	mean	±	SD
Age [years]	62.6	±	5.8
BMI [kg/m²]	28.5	±	3.7
Waist circumference [cm]	94.8	±	8.8
FPG [mmol/L]	5.49	±	0.4
HbA1c [%]	5.76	±	0.3
Insulin [pmol/L]	43.4	±	42
C-Peptide [nmol/L]	0.75	±	0.3
HOMA-IR	1.52	±	1.4
Cobalamin [pmol/L]	469	±	206
Folic acid [nmol/L]	22.3	±	6.7
Ex-smokers [%]	33.3		

	HbA1c ≤ 7.5 %			HbA1c > 7.5 %	
	Med A<br>n=16	Med B<br>n=39	Med C<br>n=19	Med B<br>n=33	Med C<br>n=37
	mean ± sd	*mean ± sd*	*mean ± sd*	*mean ± sd*	*mean ± sd*
General characteristics					
Age [y]	70.0 ± 9.5	66.8 ± 11	71.4 ± 7.0	66.8 ± 9.2	65.3 ± 11
Weight [kg]	79.2 ± 16	83.9 ± 21	89.0 ± 17	88.7 ± 16	94.4 ± 23
BMI [kg/m²]	31.4 ± 6.2	34.1 ± 8.6	34.8 ± 5.5	35.0 ± 5.9	37.6 ± 8.7
Waist circumference [cm]	97.3 ± 14	103 ± 15	107 ± 13	105 ± 12	110 ± 15
Hip circumference [cm]	114 ± 14	116 ± 16	119 ± 13	119 ± 13	123 ± 17
Waist-to-hip ratio	0.85 ± 0.0 [ab]	0.89 ± 0.0	0.89 ± 0.0	0.88 ± 0.0 [a]	0.89 ± 0.1 [b]
Blood pressure systolic [mmHg]	140 ± 23	137 ± 15	138 ± 25	146 ± 16	146 ± 20
Blood pressure diastolic [mmHg]	83.7 ± 13	82.6 ± 10	78.5 ± 12	84.9 ± 10	81.9 ± 9.5
Diabetes duration [y]	10.1 ± 6.4 [a]	14.1 ± 10	15.6 ± 6.5	13.5 ± 6.0	16.5 ± 7.8 [a]
Fasting plasma glucose [mmol/L]	6.52 ± 1.2 [abc]	8.36 ± 1.5 [ad]	8.24 ± 1.8 [e]	9.58 ± 1.5 [b]	10.6 ± 2.3 [cde]
Fasting insulin [pmol/L]	94.2 ± 85	95.0 ± 54	171 ± 138	135 ± 133	122 ± 65
C-peptide [nmol/L]	0.96 ± 0.6	1.13 ± 0.5 [a]	0.90 ± 0.6	1.33 ± 0.8 [b]	0.74 ± 0.5 [ab]
HOMA-IR	1.83 ± 1.5	2.40 ± 3.0	3.18 ± 2.0	2.62 ± 2.1	2.80 ± 1.6
Cobalamin [pmol/L]	222 ± 83	338 ± 196	326 ± 210	259 ± 111	337 ± 158
Folic acid [nmol/L]	27.2 ± 17	20.7 ± 13	18.2 ± 6.0	16.7 ± 8.8	20.1 ± 11
Total cholesterol [mmol/L]	4.46 ± 0.6	4.10 ± 0.7	3.99 ± 0.5	4.34 ± 0.8	4.60 ± 1.1
HDL cholesterol [mmol/L]	1.57 ± 0.5	1.42 ± 0.4	1.41 ± 0.4	1.31 ± 0.3	1.26 ± 0.3
LDL cholesterol [mmol/L]	2.29 ± 0.6	2.04 ± 0.5	1.78 ± 0.5 [a]	2.25 ± 0.7	2.41 ± 0.9 [a]
Triglycerides [mmol/L]	1.40 ± 1.0	1.39 ± 0.6	1.74 ± 0.8	1.71 ± 0.7	2.35 ± 2.4
Framingham risk score [%]	12.8 ± 7.6	10.8 ± 4.7	12.4 ± 6.6	14.6 ± 7.0	15.1 ± 7.7
Genomic damage					
Basal cells [‰]	9.06 ± 2.6	8.62 ± 3.0	6.95 ± 2.6	8.30 ± 4.1	9.16 ± 2.9
Micronuclei in cells [‰]	0.65 ± 0.3 [a]	0.83 ± 0.3	0.84 ± 0.2	1.04 ± 0.7	1.34 ± 0.8 [a]
Total Micronuclei [‰]	0.68 ± 0.3 [ab]	0.86 ± 0.3 [c]	0.95 ± 0.4	1.60 ± 1.3 [b]	2.06 ± 1.5 [ac]
Binucleated cells [‰]	22.4 ± 9.0	25.4 ± 9.5	26.4 ± 12.6	24.2 ± 7.9	24.6 ± 9.8
Nuclear buds [‰]	2.75 ± 1.3	2.69 ± 1.0	2.74 ± 1.0	3.10 ± 1.3	3.00 ± 1.1
Karyorrhexis [‰]	20.2 ± 7.2	26.1 ± 8.5	22.2 ± 9.0	22.8 ± 7.2	23.3 ± 6.3
Condensed Chromatin [‰]	28.1 ± 13	23.4 ± 11	27.4 ± 9.7	21.5 ± 9.6	25.2 ± 10
Karyolysis [‰]	33.1 ± 10 [a]	33.2 ± 13 [bc]	33.2 ± 14 [d]	47.8 ± 24 [b]	46.6 ± 16 [acd]
Pyknosis [‰]	1.44 ± 1.5	1.26 ± 1.0	1.21 ± 0.9	1.00 ± 0.8	1.35 ± 0.7

Table S2 Differences in general characteristics and genomic damage between Med groups divided by HbA1c threshold of 7.5%

Pairwise comparisons were tested with one-way Anova with Bonferroni adjustment or Kruskal-Wallis Test for nonparametric data. Identical letters a, b, c, d indicate significant differences between the respective groups. Significance was assumed with $p<0.05$.
